# The Dual Role of Neutrophils in Inflammatory Bowel Diseases

**DOI:** 10.3390/jcm5120118

**Published:** 2016-12-17

**Authors:** Odile Wéra, Patrizio Lancellotti, Cécile Oury

**Affiliations:** 1 GIGA-Cardiovascular Sciences, University of Liège, 4000 Liège, Belgium; owera@doct.ulg.ac.be (O.W.); plancellotti@chu.ulg.ac.be (P.L.); 2 Gruppo Villa Maria Care and Research, Anthea Hospital, 70126 Bari, Italy

**Keywords:** neutrophils, intestinal inflammation, hyper-activation, therapy

## Abstract

Inflammatory bowel diseases (IBD), including Crohn’s disease and ulcerative colitis, are characterised by aberrant immunological responses leading to chronic inflammation without tissue regeneration. These two diseases are considered distinct entities, and there is some evidence that neutrophil behaviour, above all other aspects of immunity, clearly separate them. Neutrophils are the first immune cells recruited to the site of inflammation, and their action is crucial to limit invasion by microorganisms. Furthermore, they play an essential role in proper resolution of inflammation. When these processes are not tightly regulated, they can trigger positive feedback amplification loops that promote neutrophil activation, leading to significant tissue damage and evolution toward chronic disease. Defective chemotaxis, as observed in Crohn’s disease, can also contribute to the disease through impaired microbe elimination. In addition, through NET production, neutrophils may be involved in thrombo-embolic events frequently observed in IBD patients. While the role of neutrophils has been studied in different animal models of IBD for many years, their contribution to the pathogenesis of IBD remains poorly understood, and no molecules targeting neutrophils are used and validated for the treatment of these pathologies. Therefore, it is crucial to improve our understanding of their mode of action in these particular conditions in order to provide new therapeutic avenues for IBD.

## 1. Introduction

Inflammatory bowel diseases (IBD) are characterised by chronic uncontrolled inflammation affecting the gastro-intestinal tract and leading to multiple symptoms such as weight loss, abdominal pain, recurrent diarrhoea and bleeding [1]. The two major forms are ulcerative colitis (UC) and Crohn’s disease (CD), which have distinct clinical, histopathological, endoscopic and radiological features. The etiologies and pathogenesis of these disorders clearly differ, and are not fully understood; they include complex interconnexion between enteric commensal microbiota and the host immune response, with genetic predisposition that can influence the disease development [1,2]. The aberrant immunological responses that take place in the gut can affect the epithelial barrier, increase intestinal permeability for novel antigens and further lead to a persistent chronic inflammation without tissue regeneration.

In normal conditions, the single-layered intestinal epithelium constitutes a physical and immunological barrier that prevents direct contact between luminal microbiota and intestinal mucosa. In this context, when epithelium is injured, neutrophils are crucial to protect from invading pathogens. Neutrophils can be recruited to the site of infection, recognise, phagocytose and kill pathogens by producing reactive oxygen species (ROS) with antimicrobial potential, by releasing lytic enzymes from their granules, and also by liberating neutrophil extracellular traps (NETs).

In humans, neutrophils account for 50%–70% of circulating leukocytes, whereas only 10%–25% of leukocytes are neutrophils in mice [3]. Under physiological conditions, neutrophils are cleared from the circulation in the liver, the spleen and bone marrow; these cells are absent from healthy human intestinal mucosa. When not properly eliminated, neutrophils can contribute to significant tissue damages during acute and chronic diseases. This destructive potential of neutrophils requires a tight control of their recruitment and action into tissues.

Neutrophil contribution to the pathogenesis of IBD remains controversial, and likely differs between CD and UC [4].

In experimental animal models of IBD, while some studies showed that neutrophil depletion using anti-neutrophil antibodies ameliorates colitis induced by dextran sulphate sodium (DSS) or trinitrobenzene sulphonic acid (TNBS) in the rat [5,6], others indicated a beneficial role of neutrophils during colitis, with exacerbation of inflammation after their depletion [7,8]. This discrepancy is probably due to the use of different rodent models of colitis. They induce colitis by different mechanisms, either by direct toxicity on epithelial cells and disruption of intestinal barrier integrity (DSS and TNBS) or by dysregulating immune homeostasis (transfer of CD45RB^high^ naïve T cells) (see below). It can also be due to the use of anti-Gr1 antibody that not only depletes neutrophils but also monocytes. Indeed, macrophages, concurrently with dendritic cells, have a crucial role in the limitation of neutrophil infiltration during colitis [9]. In addition, resident macrophages in the gut display a non-inflammatory phenotype and may have an immuno-suppressive role [10].

In patients, the role of neutrophils in IBD has been difficult to assess. In ulcerative colitis, the extent of neutrophil infiltration correlates with the severity of the disease, and is included in the scoring system of UC severity [11]. Neutrophil-to-lymphocyte ratio (NLR) is increased in the blood of patients with active UC compared to controls [12]. Nevertheless, NLR fails to discriminate between active and non-active UC. In addition, some neutrophil functions, such as chemotaxis and ROS production, are elevated in UC [13,14,15]. While over-activation of neutrophils seems to be central in UC, their contribution is not so clear in CD. In CD [16], some researchers postulate that patients have a predisposition to an increased activity of their innate immune system that results in intestinal inflammation, while others consider that an inadequate innate immune response can be the main cause of CD. Indeed, several studies have reported neutrophil and/or macrophage dysfunction in CD patients [17,18,19,20]. An impaired innate immune response can lead to a delayed or incomplete removal of bacterial antigens in tissues. Subsequent persistence of these foreign constituents results in a secondary uncontrolled immune response and granulomatous reaction in the gut. Another argument in favour of this paradoxical view is the association of defective neutrophil function with IBD-like diseases [21,22].

Above these considerations, controversy possibly arises from the fact that neutrophils display themselves dual roles, either beneficial for the resolution of inflammation, or detrimental when over-activated, leading to collateral tissue damage. In other words, it has become clear that both functional deficiency and hyper-reactivity of neutrophils can cause intestinal inflammation, functional neutrophils being critical to maintain intestinal homeostasis. This review will give an overview of preclinical and clinical studies that have addressed neutrophil function in IBD, focusing on how a unique cell type could exert dual roles in disease progression.

## 2. Rodent Models of Inflammatory Bowel Disease (IBD)

The relevance of experimental animal models to IBD pathogenesis has been debated [23,24]. During the last two decades, a large number of therapies for IBD emerging from preclinical investigation failed to show beneficial effects in patients. This failure is probably not surprising regarding the differences that exist between humans and animals. For instance, IBD patients can be seen as a heterogeneous population where each person carries his own combination of genetic variants, his unique gut microbiome, and has been exposed to specific environmental factors. Nevertheless, because of practical and ethical issues, animal models appear to be a reasonable alternative to experimental studies that cannot be performed in humans. For instance, mice are born and kept in a controlled environment, especially in pathogen-free conditions, and have the same genetic background. The animals allow the production of homogeneous results that are easier to interpret and reproduce. In addition, genetically engineered mice provide the advantage to dissect and individually understand the contribution of each pathway in intestinal inflammation. Animal models also give the opportunity to monitor disease development from symptomless early stages to the established pathology.

As IBDs are complex and multifactorial, rodent models cannot recapitulate completely human disease, but they can still be useful to understand pathogenesis and identify new potential therapeutic avenues. Like for any other disease, important biological differences between animals and humans cannot be ignored and must be taken into account by researchers to translate findings from the bench to bedside. Human clinical trials should still consider specific circumstances under which efficacy has been observed in animals.

In this review, we will discuss the most widely used rodent models of IBD, the chemically induced colitis models, caused by dextran sulphate sodium (DSS) or trinitrobenzene sulphonic acid (TNBS), and the adoptive transfer model in mice [24,25,26,27].

DSS-induced colitis is one of the most widely used models of IBD because of its simplicity and the high degree of uniformity and reproducibility of colonic lesions. DSS is administered in drinking water of mice or rats. Depending on the molecular weight used, the concentration, the duration, and the frequency of administration, animals will display acute or chronic colitis. Furthermore, in the same model, mice display differential susceptibilities to colitis due to genetic (strain, gender) and microbiologic variations (intestinal flora).

The mechanisms by which DSS induces colitis are not clearly elucidated. DSS is toxic for colonic epithelial cells and can induce the loss of intestinal barrier integrity, leading to an increased permeability for some molecules like DSS itself, and luminal antigens or microorganisms. This leads to an inflammatory reaction that is morphologically and symptomatically similar to the one observed in ulcerative colitis in humans.

The second compound used is TNBS. TNBS is dissolved in ethanol and administered intra-rectally in rodents. Ethanol is not only a solvent but is also needed to disrupt intestinal barrier. TNBS is considered to be a hapten that, when coupled to high molecular weight proteins, renders them immunogenic to the host immune system. Variations in the dosage of TNBS and ethanol cause differences in colitis severity. In mice, doses used usually mimic characteristics of Crohn’s disease. Finally, colitis can be initiated by adoptive transfer of syngeneic splenic CD4^+^CD45RB^high^ T cells into T and B cell-deficient recipient mice [28]. CD4^+^CD45RB^high^ T cells consist of naive T cells able to induce chronic intestinal inflammation by disruption of T cell homeostasis. In the colon, mononuclear and polymorphonuclear cell infiltration, crypt abscesses and epithelial cell hyperplasia and erosions can be observed. This model allows the analysis of the earliest immune mechanisms involved in the induction of gut inflammation as well as in the installation of a chronic disease.

## 3. Effects of Targeting Neutrophil Chemotaxis and Extravasation

Neutrophil migration into the colon mucosa is a hallmark of inflammatory bowel diseases, but still differences exist between UC and CD [29]. In UC, neutrophil accumulation in stool of patients positively correlates with active disease [30] and neutrophil infiltration is associated with the severity of the disease [11]. In contrast, using skin window chambers, several studies report a defect in neutrophil recruitment in CD patients that is not observed in UC patients [17,18,31,32]. In vitro tests show that neutrophils themselves are competent and that the decreased accumulation of these cells can be attributed to the presence of circulating inhibitors of chemotaxis in patient serum or to an inappropriate release of chemotactic mediators by resident macrophages [17,18,33]. Even if these findings seem surprising, the subsequent establishment of chronic inflammation can be explained as follows: reduced recruitment of neutrophils to sites of pathogen invasion causes persistence of bacteria into tissues and possibly within macrophages. Inefficient clearance of bacteria by macrophages drives the formation of granulomas found in CD, leading to an upregulated adaptive immune response. However, the mechanisms by which neutrophils migrate into the colonic mucosa are incompletely understood. Elucidating these pathways could provide new therapeutic opportunities to modulate neutrophil migration without eliminating it, therefore preserving host-defence.

In a general manner, extravasation of neutrophils from the vasculature to inflamed tissue follows sequential steps: tethering, rolling, adhesion, crawling, and then transmigration (reviewed in details in [3,34]).

At the site of inflammation, resident sentinel cells, such as macrophages, are activated by diverse stimuli, including pathogen-associated molecular pattern (PAMPs), and damage-associated molecular pattern (DAMPs) molecules. Then, these cells release pro-inflammatory mediators and chemokines to initiate neutrophil mobilisation and recruitment. This is first allowed by expression of adhesion molecules by the endothelium, P-, L- and E-selectins. These molecules bind their carbohydrate ligands, including P-Selectin Glycoprotein Ligand 1 (PSGL-1), leading to the tethering and then rolling of circulating neutrophils through weak and reversible interactions. In experimental colitis, P-selectins mediate rolling and recruitment of leukocytes in the colon [35]. Indeed, leukocyte rolling and adhesion in colonic venules is reduced in P-selectin-deficient mice compared to WT mice and after immune-blockage of P-selectin. This is also accompanied by a decreased neutrophil infiltration and colonic myeloperoxidase (MPO) activity [35]. The same results are obtained after blockage of PSGL-1 [36].

During rolling, neutrophils remain close to chemokines trapped by heparan sulphates on the endothelium surface [37]. These chemokines activate neutrophils and promote their firm arrest on the endothelium. For example, CXCL8 (or IL-8) in humans, and three chemokines, CXCL1, CXCL2 and CXCL5, in mice can activate neutrophils via the CXCR2 receptor. CXCR2-deficient mice display a lower susceptibility to acute and chronic colitis induced by DSS, with less inflammatory cell infiltration and less ulcer formation [38,39]. Similarly, blocking CXCR2 with antibodies or with antagonists dampens colitis [39,40], as knockdown or blockage of some other CCR and CXC receptors [41,42,43].

In humans, IL-8 is over-expressed in colonic tissues of UC patients. Its level correlates with the number of infiltrated neutrophils and with the severity of the disease [44,45]. In UC, IL-8 is not only responsible for attraction but also activation of neutrophils. IL-8 concentration is correlated with MPO concentration in colon and is responsible for an increased CD11b expression and ROS production by neutrophils [13,14]. In contrast, Marks et al. showed a defect in neutrophil recruitment associated with a reduced IL-8 production in patients with Crohn’s disease [32,46]. Neutrophil accumulation and IL-8 production are reduced in the intestine of CD patients compared to UC patients and controls. They observed the same results after subcutaneous injection of heated-killed *E. coli* in the forearm (skin window chambers) of individuals, suggesting a systemic constitutional abnormality. Addition of exogenous IL-8 into the skin restored neutrophil recruitment indicating that these cells are able to respond in the presence of an appropriate stimulus. The defect of neutrophil recruitment in CD can thus be attributed to a lower release of IL-8 by resident macrophages. Indeed, in response to pro-inflammatory stimulus in vitro, the authors demonstrated that macrophages from CD patients produce less IL-8 than control cells.

Notably, chemokines can also be released from circulating activated platelets and promote leukocyte recruitment. A significant proportion of neutrophil extravasation in inflamed colon is regulated by platelets. In addition to support monocyte recruitment to inflamed endothelia, CCL5 derived from platelets mediate neutrophil recruitment in inflamed colon [47,48]. After platelet depletion or immunodepletion of CCL5, disease activity index, tissue damage, and neutrophil infiltration is reduced in mice with acute colitis [49]. CCL5 has also been implicated in colitis development in rats [50].

Chemokines stimulate GPCR receptors and allow conformational change of neutrophil surface integrins through an inside-out signalling process [51,52]. This induces an increased affinity for their ligands on endothelium and thus firm arrest of neutrophils on it. Neutrophils mainly express integrins LFA-1 (or α1β2; CD11a/CD18) and Mac-1 (or α2Mβ2; CD11b/CD18) that can interact with ICAM-1 and ICAM-2 on activated endothelium, respectively. Along the endothelium, chemokines also create a gradient, which guides neutrophils during crawling to the preferential sites of emigration. Transmigration can be paracellular (at endothelial cell-cell junctions) or transcellular (through an endothelial cell) [53,54]. Neutrophils preferentially use the paracellular route. Transmigration also requires integrins and CAM molecules (ICAM-1/2, vascular cell adhesion protein 1 (VECAM-1), platelet-endothelial cell adhesion molecule 1 (PECAM-1)).

Cell adhesion molecules are critical for the migration of leukocytes from the circulation toward the colonic epithelium. In rats, inhibition of CD11b/CD18 (Mac-1) integrin leads to a reduction of damage score in the colon induced by TNBS, with a smaller number of ulcerations in addition to less infiltrating monocytes and leucocytes in the submucosa associated with a decrease in MPO activity [55]. In mice, the role of integrins seems to be more complex. While CD18 and CD11a null mice show a lower disease activity index during colitis, surprisingly CD11b knock-out mice exhibit enhanced DSS-induced colitis. The colons of CD18 null mice show the fewest numbers of neutrophils followed by the colons of CD11a null mice. Interestingly, absence of CD11b causes a small but significant decrease in neutrophil infiltrates in the colon but also an increase in plasma cell infiltration in response to DSS, suggesting that this molecule may influence plasma cell function during intestinal inflammation. This study demonstrates that loss of CD18/CD11a integrin blocks several adhesion pathways that are necessary for neutrophil recruitment during colitis and subsequent tissue damage, which remain more or less intact in CD11b null mice [56]. The role of ICAM-1 has been investigated too. Deletion or immunoblockage of this molecule reduces neutrophil infiltration and colonic damage during colitis in mice and rats [57,58].

In UC patients, CD11b, CD18, and ICAM-2 seem to be important for neutrophil transepithelial migration [59]. CD11b is expressed on neutrophils in contact with the colonic epithelium or in crypt abscesses whereas CD18 is expressed in epithelial basement membrane [59]. Neutrophils from UC patient seem to be less sensitive to blockage of migration with anti-CD11b monoclonal antibody toward ICAM-1 than neutrophils from control individuals. These results indicate that, in UC, neutrophils may have a constitutive change in their migratory capacities, being somehow hyper-reactive [60].

In view of potential importance of leukocyte trafficking in pathogenesis of IBD, some biomolecules have been designed with the aim to modulate leukocyte recruitment and retention into the intestine. Two current treatments that target integrins in IBD are vedolizumab and etrolizumab [61]. The first one is a monoclonal antibody directed against α4β7 integrin that provides good results for the treatment of UC and CD patients. α4β7 is expressed by leukocytes except neutrophils [62,63]. However, neutrophils can express integrin α4β1 (VLA-4) under certain inflammatory conditions, such as sepsis [64,65]. α4β1 is implicated in the rolling of neutrophils along the endothelium and thus can affect the latter stage of transmigration [66]. The monoclonal antibody targeting the α4 integrin subunit, natalizumab, has demonstrated a good response with improved clinical remission in CD treatment [67].

While neutrophil accumulation is increased in UC, CD seems to result from an impaired recruitment of these cells. In this context, stimulating immune system rather than suppressing it may calm the subsequent excessive adaptive response. Defective neutrophil recruitment resulting from alterations in macrophage function can be counteracted by granulocyte-macrophage colony-stimulating factor (GM-CSF) administration [68]. Based on this postulate and on beneficial effects of GM-CSF therapy in glycogen storage disease type Ib, a hereditary metabolic disorder characterised by neutrophil dysfunction and intestinal inflammation, clinical trials with GM-CSF have been undertaken in CD [69,70,71]. While this treatment has been shown to be safe and effective, Roth et al. report no difference with placebo induction on clinical remission and improvement of active CD [72]. However, further investigations are required due to limited numbers of patients who have been randomised in the explorative studies, and to important heterogeneity in results between trials in addition to the lack of data on patient outcomes.

## 4. Role of Neutrophil-Derived Molecules

The presence of neutrophils is crucial for innate immunity and resistance to pathogens, as illustrated by patients with congenital or acquired abnormalities in neutrophil number and function suffering from recurrent infections [73]. Neutrophils harbour different strategies to attack invaded microorganisms: phagocytosis, release of reactive oxygen species (ROS) or soluble antimicrobials (including granule proteins), and generation of neutrophil extracellular traps (NETs). They are also able to secrete pro-inflammatory mediators affecting their own action but also other leukocyte functions.

**Figure 1:**
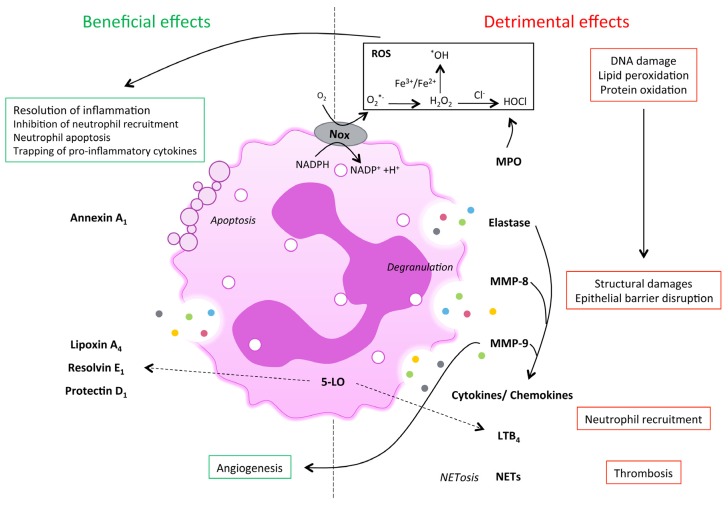
Dual role of neutrophils in intestinal inflammation. Excessive or prolonged neutrophil activation can lead to chronic inflammation in inflammatory bowel disease (IBD). Reactive oxygen species (ROS) production causes damage to DNA, lipids and proteins, altering their function. Paradoxically, ROS is also essential for the maintenance of intestinal homeostasis. Upon neutrophil activation, some proteases such as elastase, MMP-8 or MMP-9 are produced, leading to structural tissue damage and amplification of the inflammatory response through release of pro-inflammatory cytokines and chemokines from extra-cellular matrix (ECM). The enzyme 5-lipoxigenase (5-LO) is involved in the synthesis pro-inflammatory lipid mediators like LTB_4_ as well as in the production of pro-resolving lipid mediator generation, including lipoxin A_4_, resolvin E_1_ and protectin D_1_, during the resolution of inflammation. Annexin A_1_ is expressed by apoptotic neutrophils and triggers neutrophil apoptosis, neutrophil engulfment by macrophages and negatively regulates neutrophil transmigration. Finally, neutrophils can be responsible for increased risk of thrombosis in IBD through neutrophil extracellular traps (NET) release. Abbreviations: Nox: NADPH oxidase; ROS: reactive oxygen species; MPO: myeloperoxidase; MMP: matrix metalloprotease; LTB_4_: leukotriene B_4_; NETs: neutrophil extracellular traps.

There are three types of granules formed consecutively during neutrophil maturation. Primary or azurophilic granules comprise myeloperoxidase (MPO), neutrophil elastase (NE), cathespin G and lysozyme. Secondary or specific granules are composed of a wide variety of different components such as lactoferrin and collagenase (i.e., MMP-8). Finally, tertiary granules contain, for instance, gelatinase B (i.e., MMP-9). These factors have either beneficial effect when released at the right place, and at the right moment, or detrimental effect when pathological mechanisms modify their mode of action (Figure 1 and Table 1). Interestingly, the study by Dwarakanath et al. [74] reported an increased ratio of faecal lactoferrin to myeloperoxidase in IBD, but not in patients with infective diarrhoea, suggesting the occurrence of relatively selective triggering of secondary granule responses in IBD, possibly via bacterial peptides.

### 4.1. Reactive Oxygen Species

When activated, neutrophils secrete reactive oxygen species (ROS). ROS are small molecules, including the oxygen radicals (superoxide anion (O_2_**^−^**) and hydroxyl (•OH)) and non-radicals such as hypochlorous acid (HOCl), singlet oxygen (^1^O_2_) and hydrogen peroxide (H_2_O_2_). Oxygen radicals are unstable and can react with proteins, lipids or DNA. NO-derived compounds, called reactive nitrogen metabolites (RNMs) can also be formed and damage a lot of cell and tissue components. For example, peroxynitrite, resulting from the reaction between superoxide and NO, is extremely reactive. To counteract any harmful effects of ROS, tissues such as intestinal mucosa possess an efficient antioxidant protection system. Superoxide can be converted to more stable H_2_O_2_ by superoxide dismutase (SOD) in the mitochondria. In turn, H_2_O_2_ can be transformed in water and O_2_ by catalase or in water by glutathione peroxidase. H_2_O_2_ can also react non-enzymatically with O_2_**^−^** to form •OH during the Fenton reaction in the presence of Fe^2+^.

IBDs are characterised by an imbalance between the production of ROS and antioxidants, thereby affecting gut homeostasis [75,76]. Dysfunction of mechanisms that regulate levels of ROS production can lead to persistent inflammation in intestinal tissue. Prolonged production of high concentrations of ROS causes DNA damage, lipid peroxidation and protein oxidation, altering the function of these molecules. For example, ROS can degrade polyunsaturated acids within the membrane of intestinal epithelial cells, which results in disruption of cell membrane and increased mucosal permeability [77]. Lipid peroxidation is significantly increased in colonic mucosa during DSS or TNBS treatment, suggesting that the induction of lipid peroxidation is an early critical event in these experimental models of IBD [77].

In support of an ROS deleterious effect, treatment of mice by free radical scavengers or antioxidants reduces the severity of chemically induced colitis [78]. Likewise, administration of a superoxide dismutase/catalase mimetic nanomedicine comprising a hydrogen peroxide-eliminating nanomatrix and a free radical scavenger (Tempol) suppresses the expression of pro-inflammatory mediators in different mouse models of colitis [79]. In rats, treatment with different doses of SOD attenuates colonic tissue damages and lipid peroxidation in a dose dependent manner and reduces rolling and adhesion of leukocytes in venules [80]. Alpha lipoic acid, as a potent antioxidant and anti-inflammatory agent, reduced plasma levels of lipopolysaccharides (LPS) and systemic inflammation in mice with colitis [81].

ROS production is augmented in colonic mucosa of patients with IBD [76]. In both CD and UC patients, an imbalance between the different antioxidant enzymes coupled with an inefficient endogenous antioxidant response results in an increased formation of ROS and RNMs within the colon. However, to date, there is little evidence that these species contribute to the pathogenesis and the maintenance of inflammatory process in patients [82]. The level of malondialdehyde (MDA), an end product of lipid peroxidation, is higher in CD and UC mucosa compared to non-inflamed and control mucosa [76]. In the same study, 3-nitro-l-tyrosine (3-NT), an index of peroxynitrite-mediated protein nitration expressed by lamina propria neutrophils, was significantly higher in UC than in CD. Notably, a defect in neutrophil respiratory burst and reduced cell viability are also observed in CD patients [83]. This may be related to a decreased defensive potential of neutrophils against luminal microbes, leading to the recruitment of other inflammatory cells, and to the establishment of chronic inflammation [84]. Importantly, in addition to deleterious effect, ROS can mediate appropriate healing responses. In particular, production of ROS by neutrophils can create a hypoxic niche by oxygen consumption, which may aid in the resolution of inflammation [85]. Moreover, ROS generated by NADPH oxidase are also important for neutrophil apoptosis, and subsequent restoration of tissue homeostasis [86].

The importance of physiological levels of ROS in the gut is also highlighted by chronic intestinal inflammation affecting patients with inactivating gene defects in NADPH oxidase [22,87]. Chronic granulomatous disease (CGD) is an inherited immunodeficiency disorder caused by inactivating mutations in the genes encoding the NOX-2 complex, one of the NADPH oxidase (NOX) isoforms. This disease is characterised by recurrent bacterial and fungal infections that are often life-threatening. These patients display abundant granulomas in a lot of organs, including the intestine. Granulomatous colitis is similar to the one observed in CD patients [22,87].

NOX-2 oxidase is mainly expressed by neutrophils. It comprises different sub-units, including p40^phox^, p47^phox^, p67^phox^, forming a complex in the cytosol, plus p22^phox^, and gp91^phox^, located in the membranes of secretory vesicles and specific granules [76]. The role of NOX-2 in IBD development seems to be complex as p47^phox^ knock-out mice do not spontaneously develop colitis, but they display increased susceptibility to DSS colitis, while mice lacking p91^phox^ develop less severe colitis than WT mice following DSS treatment [88,89]. NOX-1, which is often called “colon NADPH oxidase” because of its high expression in colonic epithelium, is also required for healing after colitis [90].

ROS can also be generated by mitochondria (mtROS). During energy production, ATP molecules are produced thanks to an electrochemical proton gradient maintained by the transfer of electrons to protein carriers in the mitochondrial membrane called Complex I–IV. During this process, electrons can leak out from the electron transport chain and be aberrantly transferred to O_2_ to form superoxide radicals. Increased mtROS levels are observed in IBD patients, and decreasing mtROS dampens colitis [91].

### 4.2. Matrix Metalloproteases

After passing through the endothelial cell layer, neutrophils have to cross the basement membrane to progress into tissue and reach the inflammatory site. Neutrophils express proteases, including serine proteases (like elastase), and matrix metalloproteases (MMPs) that degrade the extracellular matrix (ECM) [3,92]. MMPs are involved in cellular matrix turnover during normal growth, development and reproduction. These proteases also permit the release of cytokines, and control their concentration into tissue concurrently to the regulation of the activity of ECM-associated chemokines.

MMP-9, also known as gelatinase B, is one of the main MMP secreted by neutrophils. In MMP-9 knock-out mice, DSS-induced colitis and the associated increase of intestinal permeability are attenuated [93]. Accordingly, selective inhibition of MMP-9 reduces disease severity in mice [94]. However, the use of total knock-out animals does not allow to discriminate the role of neutrophil MMP from MMP expressed by other cell types. Indeed, it has been demonstrated that epithelial and not neutrophil-derived MMP-9 mediates tissue damage during colitis [95]. Notwithstanding, a detrimental effect of neutrophil MMP-9 has been shown in several inflammatory diseases like COPD, as well as in stroke and cancer propagation [96,97,98]. During the inflammatory process, degradation of collagen by MMP-9 contributes to chronic neutrophilic infiltration [99]. In inflamed intestine, combined action of MMP-8, MMP-9 and prolyl oligopeptidase (PE) allows the release of collagen-derived fragments, proline-glycin-proline (PGP), that have an important chemotactic effect on neutrophils. Neutrophils recruited can then release more MMPs and generate more PGPs, establishing a vicious circle that exaggerates the disease [100]. In the same manner, neutrophils can potently affect the inflammatory conditions through the modification of cytokines and chemokines that have important roles in the recruitment of additional effector cells. For instance, neutrophil-derived MMP-9 is able to cleave CXCL-8 rendering it 10- to 27-fold more effective in neutrophil activation, while cleavage of platelet factor 4 (PF4) and CXCL-1 leads to their inactivation [101]. Neutrophil-derived MMP-8, also known as collagenase, can also modulate the activity of several chemokines [102]. The activation of these chemokines promotes more neutrophil infiltration and propagates the inflammation. Thus, increased levels of MMPs can not only mediate structural damage to the tissue, but they may also propagate an excessive immune response.

In IBD patients, reports have demonstrated an increased level of several MMPs in inflamed intestine, which is often accompanied by an insufficient level of the endogenous MMP inhibitors such as tissue inhibitor of metalloproteinases (TIMPs) [103,104,105]. The activity of some MMPs (MMP-1, 2, 3 and 9) is also augmented in colon of IBD patients and is correlated with MPO level and tissue damage [106]. Strong associations are shown between faecal MMP-9 and clinical, endoscopic, and histologic activities of UC, but no correlation are found between this biomarker and activity indices of CD [107].

In mice and rats treated with DSS or TNBS, immune infiltration is associated with an upregulation of MMPs, and the mucosal damage can be reversed by application of MMP inhibitors [108,109,110]. While some of these drugs show signs of efficacy in patients, all MMP-targeted inhibitors have been unsuccessful because of dose-limiting side effects and/or insufficient clinical benefit, probably due to their lack of specificity [111,112]. In UC patients, administration of a fully humanised anti-MMP-9 monoclonal antibody causes clinical remission in 14% of cases [113].

Paradoxically, MMPs can also have beneficial effects within tissues. MMP-9 itself activates pro-angiogenic vascular endothelial growth factor (VEGF), which promotes revascularisation at injured site [114,115,116]. MMPs produced by other cells are also important to resolve inflammation. MMP-10, that is produced predominantly by infiltrating myeloid cells in both murine and human colitis, is required for resolution of chronic colitis [117]. In addition, absence of MMP-19 leads to severe tissue destruction, and failure to resolve inflammation, which is attributed to a delayed but sustained neutrophil infiltration associated with higher MMP-2 and MMP-9 activities [118].

### 4.3. Neutrophil Elastase

Like MMPs, neutrophil elastase (NE) is able to degrade collagen, elastin and fibronectin, and thus, can release mediators trapped in ECM. In addition, elastase from neutrophils degrades TIMPs and activate MMPs, and so may contribute to the imbalance between these two types of enzymes observed in IBD [119]. Elastase has mainly been studied in UC. The enzymatic activity of NE is elevated in both plasma and colon of UC patients compared to healthy controls, as in mice under DSS treatment [120]. NE released in situ may impair mucosal repair through inhibiting epithelial cell proliferation in patients with UC [121]. NE may also disrupt the epithelial cell monolayer by degrading E-cadherins and zonula occludens-1 during transmigration, a process that can participate in the loss of intestinal barrier [122]. However, there is no clear evidence that NE contributes to IBD pathophysiology.

A NE-specific inhibitor, ONO-5046, reduces ulceration and inflammatory cell infiltration in the colon of DSS-treated mice [120]. Despite the fact that therapeutic inhibition of NE has demonstrated promising results in preclinical models of inflammatory bowel diseases, and in other models where this enzyme has an important implication (for instance, inflamed lung and ischaemia-reperfusion injury), translation to positive results in clinical trials remains challenging due to the complexity of regulatory mechanisms of elastase activity, and its interactions with endogenous inhibitors in disease conditions [123].

### 4.4. Other Pro-Inflammatory Mediators Released by Neutrophils

Besides MMPs, neutrophils release a lot of extracellular mediators that amplify neutrophil response in an autocrine and paracrine manner [124]. During their activation, neutrophils produce several cytokines and chemokines [125]. One of most abundant chemokines secreted by neutrophils is IL-8. As mentioned before, the levels of this potent chemokine correlate with the number of neutrophils infiltrated in the colon of UC patients, while its production is impaired in CD resulting in reduced chemotaxis [18,44,45]. Another potent chemoattractant and activator of neutrophils that is released by neutrophils themselves is CXCL-1 (also known as GRO-α). This chemokine is elevated in colonic tissue of UC patients compared to controls [126]. The CXCL-1-induced recruitment of neutrophils also plays a protective role since mice lacking this molecule display exaggerated colitis after DSS treatment [127]. The authors suggest that blocking a single chemokine may affect beneficial neutrophil recruitment that is required for the maintenance and the restoration of mucosal homeostasis. Neutrophils are able to secrete several other chemokines involved in monocyte recruitment, such as CCL-3 (MIP-1α) or CCL-4 (MIP-1β). Thus, neutrophil-derived chemokines can participate in the amplification of the innate and adaptive immune responses that take place in IBD, and in other immune-mediated inflammatory diseases [125].

Importantly, neutrophils produce pro-inflammatory cytokines that have a central role in the pathogenesis of IBD, like TNF-α. TNF-α levels are increased in the mucosa of IBD patients. It contributes to redox imbalance by inducing the expression of some NADPH oxidase isoforms within the colon, and in particular, infiltration of NOX-2-expressing cells such as neutrophils [128,129]. Anti-TNF-α therapy (infliximab) is currently used in IBD patients, as it shows beneficial effect both in CD and UC [130].

Neutrophils are also a source of lipid pro-inflammatory mediators. Leukotriene B4 (LTB4) is one of the best examples of the feedback amplification response induced by neutrophils themselves. This factor is synthetised through the action of the enzyme 5-lipoxygenase (5-LO) [131]. LTB4 is involved in neutrophil recruitment, particularly the second wave of recruitment, in different conditions such as dermatitis and arthritis [132,133,134]. In IBD patients with active disease, some enzymes involved in leukotriene pathway are significantly increased in colonic mucosa, and mice lacking 5-LO display reduced colitis induced by TNBS, in association with a decreased colonic neutrophil infiltration [135,136]. Nevertheless, while LTB4 has been demonstrated to be an important stimulus for neutrophil chemotaxis in IBD, with the chemotactic response being higher in UC than in CD, other studies have shown a decreased neutrophil sensibility to LTB4 in both UC and CD [137,138]. In accordance with this latest finding and with the defect function of neutrophils in CD, the number of LTB4 receptors in neutrophils from CD patients has been shown to be reduced compared to healthy controls contributing to a depressed chemotaxis in response to this lipid mediator [139].

Thus, understanding the mechanisms causing an excessive neutrophil activity, in particular through amplification activating loops, may be relevant to find new therapeutic targets in chronic IBD disorders.

## 5. Contribution of Neutrophils to IBD-Associated Thrombo-Embolic Events

Inflammatory bowel diseases are associated with a hyper-coagulant state and an increased risk of thrombo-embolic events [140,141]. Thrombosis is an extra-intestinal complication that can significantly contribute to the morbidity and mortality in IBD patients. The pathogenesis of thrombosis in these patients seems to be multifactorial and is not fully elucidated. It is believed that neutrophils may have an important role in these events due to their ability to promote thrombus formation.

Indeed, it has been demonstrated that neutrophils are essential for thrombus formation in a deep vein thrombosis model in mice [142]. Furthermore, in mouse arterioles, they are the first cells to be recruited to the activated endothelium, where they promote platelet activation and fibrin generation [143]. Neutrophils can promote thrombus propagation by different ways. Maugeri et al. have demonstrated that they express tissue factor (TF) upon activation [144]. In a mouse model of laser-induced injury in cremaster muscle arterioles, neutrophils represent the main source of TF at the site of injury [143]. TF is also expressed on microparticles (MPs) derived from monocytes or neutrophils. In addition, neutrophils can produce two pro-coagulant enzymes, elastase and cathepsin G. Upon FeCl_3_-induced vessel injury, these enzymes are responsible for the cleavage, and thus, inactivation of the tissue factor pathway inhibitor (TFPI) [145]. All these processes promote coagulation cascade activation and thrombin generation that, in turn, activate platelets.

In mice, TF contributes to the pathogenesis of intestinal inflammation and to the associated thrombotic events. Indeed, DSS treatment leads to enhanced thrombus formation in cremaster muscle arterioles, and to an elevation of plasma levels of thrombin-antithrombin (TAT) complexes, all of these events being reduced by TF inhibition [146]. TF inhibition also causes a decrease of the numbers of adherent platelets and leukocytes along colonic venules and less tissue damage.

Neutrophils also participate in thrombus formation by releasing neutrophil extracellular traps (or NETs). This process, called NETosis, is usually initiated by ligand binding to neutrophil toll-like receptors and receptors for IgG–Fc complexes [146]. NETs are made of chromatin fibres that serve as a platform for granular proteins such as neutrophil elastase, cathespin G, myeloperoxidase or high mobility group protein B1 (HMGB1). These components are responsible for NETs’ pro-inflammatory and pro-coagulant properties. Hence, NETs trap pathogens and prevent their dissemination by exposing them to a high concentration of anti-microbial factors, but are also able to induce platelet adhesion, activation and aggregation [131]. NETs are also involved in the thrombosis process in both veins and arteries in vivo [142,145,147].

One proteomic study has revealed that some proteins associated with neutrophils and NETs, including MPO, NE, MMP-9 or cathepsin G, are increased in biopsies of UC patients compared to controls [148]. These data have been validated by microscopy and show the presence of NETs in UC colon tissues.

Recently, it has been demonstrated that neutrophils from IBD patients release more NETs than controls [149]. The authors propose that NETs, and also the elevated phosphatidylserine (PS) exposure on MPs from different cell origins, could be the link between IBD and the hypercoagulable state observed in these conditions. In the same study, treatment of control neutrophils with sera from patients with active disease leads to elevated NET release. This suggests that these patients possess a serum microenvironment that is able to induce neutrophil-derived NETs. This could be due to anti-neutrophil cytoplasmic antibodies (ANCAs) found in serum of patients [150]. ANCAs can indeed be directed against NET components, such as MPO, NE, cathespin G or proteinase 3. Thus, as also observed in systemic lupus erythematous (SLE), ANCAs may trigger the release of NETs from neutrophils and participate in the amplification of ANCA generation by exposing new antigenic proteins [149,151].

Importantly, the capacity of neutrophils to promote thrombosis can also be associated with bleeding events. This idea is well illustrated in sepsis where an excessive coagulation activation (disseminated intravascular coagulation) leads to platelet and coagulation factor consumption, responsible for subsequent haemorrhages [152]. In IBD, patients display gastro-intestinal bleeding resulting in anaemia. However, the etiology of these bleeding events is not elucidated [153].

It has been demonstrated that neutrophils are responsible for bleeding in thrombocytopenia [154,155]. Furthermore, a higher neutrophil-to-lymphocyte ratio (NLR) is associated with a poor prognosis in patients presenting with intra-cerebral haemorrhage, or with symptomatic intracerebral haemorrhage after rtPA treatment for ischemic stroke [156,157]. Neutrophils could also be responsible for bleeding in the gastro-intestinal tract. Indeed, an increased number of macrophages and neutrophils is observed in the ulcer margin in patients with stomach ulcer that undergoes re-bleeding [158]. In UC, NLR correlates with disease severity calculated by a score comprising the extent of intestinal bleeding [159]. In addition to their pro-thrombotic phenotype, NETs also contribute to small vessel vasculitis that can result in tissue damage and subsequent haemorrhages [151].

Thus, though not demonstrated yet, neutrophils could contribute to both gastro-intestinal bleeding and pro-thrombotic phenotype in IBD patients.

## 6. Essential Role of Neutrophils in the Resolution of Inflammation

Despite neutrophils being the first immune cells recruited to the site of inflammation, their activity needs to be tightly regulated to limit collateral damage to the tissue and avoid evolution toward chronic diseases [160,161,162].

Neutrophils are regarded as short-lived cells with a half-life in the circulation of around 1.5 h in mice and 8–20 h in humans [3,160]. However, recent data suggest that their circulatory lifespan can be up to 12.5 h, and 5.4 days in healthy mice and humans, respectively [163]. Even if these results are a matter of controversy [164], it is clear that an increased neutrophil lifespan is observed under inflammatory conditions, which contributes to an efficient clearance of invading pathogens [165]. After completing their action, neutrophils normally undergo apoptosis; apoptotic neutrophils are ingested by macrophages during a process called efferocytosis. In addition, neutrophil efferocytosis leads to macrophage polarisation to a M2 phenotype [166,167]. Abnormally prolonged neutrophil lifespan due to a reduced apoptotic capacity is observed in chronic inflammation, including IBD, and results in increased disease severity.

Dying cells express *find me* signals to attract scavengers and then *eat me* signals, surface markers that allow their identification. One of the important *eat me* signals expressed by neutrophils is Annexin A1 (AnxA1). This molecule increases neutrophil engulfment by macrophages [168,169]. Importantly, AnxA1 also contributes to the resolution of inflammation by negatively regulating neutrophil transmigration, and exogenous administration of AnxA1 causes neutrophil apoptosis in inflamed lungs of humans and mice [170,171,172,173]. With all these properties, it is not surprising that AnxA1-deficient animals exhibit increased susceptibility to DSS-induced colitis, with larger mucosal injury [174]. This anti-inflammatory protein is also needed for efficient anti-TNF-α treatment, as shown by the prevention of DSS-induced rectal bleeding, diarrhoea, epithelial damage, and collagen degradation following infliximab treatment only in WT but not in AnxA1 knock-out mice [175]. A reduction of AnxA1 protein plasma levels is observed in IBD patients, and their expression is upregulated during anti-TNF-α therapy in patients with a successful intervention but not in clinical non-responders [176].

Neutrophils also actively contribute to the restoration of tissue homeostasis by secreting growth factors, such as VEGF, and pro-resolving lipid mediators. During inflammation, a switch in lipid mediator profile occurs. As mentioned before, neutrophils release pro-inflammatory lipid mediators (for example, prostaglandin I2 and LTB4) at the onset of inflammation, before producing pro-resolving lipid mediators, such as lipoxins, resolvins and protectins [177]. Lipoxin A4 (LXA4) is an anti-inflammatory agent that inhibits neutrophil recruitment and their transepithelial migration [177,178]. Patients with severe UC display lipoxin biosynthesis deficiency in the colon, characterised by low to absent synthesis of LXA4 [179]. Accordingly, lipoxin A4 analogues dampen colitis induced by TNBS or DSS [180,181]. Similar to LXA4, resolvin E1 reduces neutrophil transepithelial migration, and treatment with resolvin E1 reduces damage after TNBS administration [182,183]. These mediators, including protectin D1, promote phagocytosis of apoptotic neutrophils [184]. They also mediate the upregulation of CCR5 receptors on apoptotic neutrophils that sequester pro-inflammatory chemokines such as CCL3 and CCL5, leading to neutrophil clearance at sites of inflammation [185].

Finally, neutrophils secrete proteins that have important functions in modulating the inflammatory response, and preventing undesired tissue damage. Pentraxin (PTX)3, a member of the PTX family, is stored in neutrophil granules and is released in response to microbial recognition [186]. Upon release, PTX3 also co-localises with NETs. This protein has a pro-inflammatory role by activating complement cascade and stimulating opsonisation [187,188]. However, PTX3 can also bind to P-selectin and impair neutrophil rolling and recruitment in vivo [189]. Mice lacking PTX3 exhibit increased leukocyte rolling in thrombin-stimulated mesenteric venules, and increased leukocyte recruitment in a model of acute lung injury [189]. Neutrophils are the main source of PTX3 in UC patients and their expression levels correlate with the histological grade of inflammation [190]. Plasma levels of PTX3 are also higher in patients with active disease than in normal subjects and patients with inactive disease [191].

Thus, by inhibiting their own recruitment and promoting their own removal, neutrophils contribute to the resolution of inflammation and tissue repair. Neutrophil clearance by itself allows the inflammation resolution by reducing the number of pro-inflammatory cells. However, apoptotic neutrophils are also required for proper resolution. This indicates that simply depleting neutrophils would not be protective against intestinal inflammation since the action of neutrophils is mandatory to clear infection, to avoid runaway immune responses, and to restore tissue homeostasis.

Therapeutic strategies that promote neutrophil apoptosis have emerged in different inflammatory models, but, to date, no such molecules are used and validated for treatment of IBD [160]. We can postulate that a long-term treatment that induces neutrophil apoptosis or shortens neutrophil life span during inflammation may attenuate detrimental innate immune responses.

## 7. Conclusions

In summary, though not fully elucidated yet, the role of neutrophils in the pathogenesis of IBD would be dual. It may differ between Crohn’s disease (CD) and ulcerative colitis (UC). In UC, unrestricted neutrophil activation may cause significant tissue damage that further leads to chronic pathology, whereas in CD, defective neutrophils may not be able to limit invasion by microorganisms, leading to subsequent uncontrolled inflammatory reaction. Each component brought by neutrophils are essential for their normal action, and they can also trigger positive feedback amplification loops that promote neutrophil recruitment and activation, contributing to the pathogenesis of several diseases such as autoimmune and chronic diseases [124,192]. In addition, some neutrophil-derived factors can be detrimental by inducing tissue damage, while they can also be essential to maintain tissue homeostasis. This concept is illustrated by chronic granulomatous disease (CGD) patients who display inflammatory bowel disease (IBD)-like disease, even though reactive oxygen species (ROS) production is often related to DNA damage, lipid peroxidation, and protein oxidation, as well as to the aging process. Another example is matrix metalloprotease-9 (MMP-9), which is required for angiogenesis and tissue regeneration, but can also create important structural damage, and promote a second wave of neutrophil recruitment at a later stage of the inflammatory process. Importantly, neutrophils also actively participate in the resolution of inflammation. All these considerations can explain some controversial observations when using different agents blocking neutrophil action during colitis. Consequently, drugs that respect neutrophil normal action while limiting their “hyper-action” may be preferred to full neutrophil depletion.

Finally, the duality in neutrophil action during colitis may also be explained by the appearance of distinct neutrophil populations in diseased conditions. Indeed, like other immune cells, neutrophils are a heterogeneous population of cells comprising pro-inflammatory and anti-inflammatory neutrophils. Different neutrophil subsets have been observed in cancer, where anti- (N1) and pro-tumoral (N2) neutrophils can be found, but also in autoimmune diseases like lupus and Methicillin-resistant Staphylococcus aureus infection in mice [193,194,195,196]. Thus, from short-lived cells with limited capacities, neutrophils emerge to be a heterogeneous population with prolonged lifespan and functional versatility. Under hypoxic conditions, a specific subset of MMP-9 delivering neutrophils can be recruited to promote angiogenesis [115]. Some subsets may also display the ability to supress T cell responses, which can be critical in the pathogenesis of IBD [197].

Therefore, the understanding of the mechanisms governing neutrophil activity, and the origin and function of different neutrophil subsets all require further investigations in order to provide new therapeutic avenues for IBD.

## Figures and Tables

**Table 1:** Summary of beneficial and detrimental roles of factors brought by neutrophils in inflamed tissues.

	Beneficial Roles	Detrimental Roles
NADPH oxidase (ROS)	Pathogen killing	Epithelial barrier disruption and structural damages (lipid peroxidation in epithelial cell membrane)
	Resolution of inflammation (neutrophil apoptosis)	
MMPs	Angiogenesis in hypoxic tissue	Amplification of inflammatory response and subsequent neutrophil recruitment
Elastase		Epithelial barrier disruption and structural damages (degradation of E-cadherins and zonula occludens-1)
		Thrombosis (inhibition of TFPI)
5-lipoxygenase	Resolution of inflammation Pro-resolving lipid mediator generation (lipoxin A_4_; resolvin E_1_; protectin D_1_)	Amplification of inflammatory response Pro-inflammatory lipid mediators generation (LTB_4_)
NETs	Pathogen killing	Thrombosis (pro-thrombotic components)
